# ‘Spikelet stop’ determines the maximum yield potential stage in barley

**DOI:** 10.1093/jxb/erab342

**Published:** 2021-07-22

**Authors:** Venkatasubbu Thirulogachandar, Thorsten Schnurbusch

**Affiliations:** 1 Independent HEISENBERG Research Group Plant Architecture, Leibniz Institute of Plant Genetics and Crop Plant Research (IPK), OT Gatersleben, SeelandGermany; 2 Institute of Agricultural and Nutritional Sciences, Faculty of Natural Sciences III, Martin Luther University Halle-Wittenberg, D-Halle, Germany; 3 CSIRO Agriculture and Food, Australia

**Keywords:** Barley, grain yield, inflorescence meristem, maximum yield potential, spikelet initiation, spikelet growth

## Abstract

Determining the grain yield potential contributed by grain number is a step towards advancing the yield of cereal crops. To achieve this aim, it is pivotal to recognize the maximum yield potential (MYP) of the crop. In barley (*Hordeum vulgare* L.), the MYP is defined as the maximum spikelet primordia number of a spike. Many barley studies assumed the awn primordium (AP) stage to be the MYP stage regardless of genotypes and growth conditions. From our spikelet-tracking experiments using the two-rowed cultivar Bowman, we found that the MYP stage can be different from the AP stage. Importantly, we find that the occurrence of inflorescence meristem deformation and its loss of activity coincided with the MYP stage, indicating the end of further spikelet initiation. Thus, we recommend validating the barley MYP stage with the shape of the inflorescence meristem and propose this approach (named ‘spikelet stop’) for MYP staging. To clarify the relevance of AP and MYP stages, we compared the MYP stage and the MYP in 27 barley accessions (two- and six-rowed accessions) grown in the greenhouse and in the field. Our results reveal that the MYP stage can be reached at various developmental stages, which greatly depend on the genotype and growth conditions. Furthermore, we propose that the MYP stage and the time to reach the MYP stage can be used to determine yield potential in barley. Based on our findings, we suggest key steps for the identification of the MYP stage in barley that may also be applied in a related crop such as wheat.

## Teaser

The barley maximum yield potential (MYP) stage can differ from the awn primordium stage and varies depending on the genotype and growth conditions. We suggest key steps to identify MYP in barley that might apply to related cereals.

## Introduction

Grain yield potential is the key to augmenting the yield of our crops that is needed for the subsistence of humanity. In the small-grain cereals, such as wheat (*Triticum* spp.) and barley (*Hordeum vulgare*, L), grain number (GN) per unit area shows a significant influence on the final grain yield (Prystupa *et al.*, 2004; Arisnabarreta and Miralles, 2006*b*, 2015; Peltonen-Sainio *et al.*, 2007; Ugarte *et al.*, 2007; Serrago *et al.*, 2013; García *et al.*, 2015). Wheat and barley belong to the tribe Triticeae and prototypically possess an unbranched inflorescence known as the spike. The spike of these crops follows a distichous phyllotactic arrangement of spikelets (basic floral units of the spike) on its rachis (spike axis), whereas, architecturally, the spike apex (inflorescence meristem) and spikelets are different in these crops. In wheat, the spike is determinate; that is, spikelet initiation culminates by formation of a terminal spikelet; however, the barley spike is indeterminate and produces spikelets indefinitely without a terminal spikelet. Furthermore, wheat develops a single spikelet on every rachis node, and its spikelet produces florets (reduced flowers) indeterminately. In contrast, barley forms three spikelets at every node, while its spikelets are determinate and bear a single floret (Bonnett, 1935, 1936; Whipple, 2017; Bommert and Whipple, 2018; McKim *et al.*, 2018; Gauley and Boden, 2019; Koppolu and Schnurbusch, 2019).

In wheat and barley, GN is mostly determined by the development and survival of the florets and spikelets (Arisnabarreta and Miralles, 2008*a*, 2015; Bancal, 2008, 2009; Gonzalez *et al*., 2011; Ferrante *et al.*, 2013*a*, *b*; Digel *et al.*, 2015; Sakuma *et al.*, 2019; Sakuma and Schnurbusch, 2020). To determine the surviving portion of the florets/spikelets, it is necessary to identify the maximum number of florets/spikelets, also popularly known as ‘maximum yield potential’ (MYP). In wheat, MYP is ascertained by tracking the floret number of spikelets (indeterminate) located at various spike positions (basal, central, and apical) until the spikelets cease floret initiation (Gallagher, 1979; Ferrante *et al.*, 2013*b*, 2020; Guo and Schnurbusch, 2015). To define the MYP in barley, the spikelet/floret number per spike needs to be followed up until the stage at which the indeterminate barley spike ceases spikelet initiation (Gallagher *et al.*, 1976; Kirby, 1977; Appleyard *et al.*, 1982; Arisnabarreta and Miralles, 2006*a*; Arduini *et al.*, 2010). The above-mentioned studies indicated that the MYP must be discerned by following the indeterminate floral structure (spikelet or spike) until it stops its activity. By enumerating the spikelet primordia number from sowing until anthesis, Appleyard *et al.* (1982) conducted a study using three barley genotypes, including their crosses. They identified the maximum number of spikelets after spikelet initiation had stopped (fig. 1 in Appleyard *et al.*, 1982). The authors found that the number of spikelets counted at the MYP stage was close to the spikelet number at the awn primordium (AP) stage (Kirby and Appleyard, 1984) since the average difference was only 0.52 primordium. It seems astonishing, but in fact many subsequent studies presumed that barley’s MYP is around the AP stage irrespective of the genotypes and the growth conditions used in such experiments (Kirby and Appleyard, 1984; del Moral *et al.*, 1991; Miralles *et al.*, 2000; Arisnabarreta and Miralles, 2006*a*, 2010, 2015; Alqudah and Schnurbusch, 2014). This initial assumption appears simplistic and premature, and thus requires more experimental verification to better understand the relevance of the AP stage corresponding to the MYP using various barley accessions and growth conditions.

In the present study, we therefore provided a comprehensive description of variations for MYP tested in different genotypes and growth conditions. First, we analyzed the MYP stage on the main culm of a two-rowed cv. Bowman grown in seven different experiments (variable growth conditions and pot sizes). In these experiments, we identified the MYP stage by following the spikelet number per spike until the cessation of spikelet initiation. Among the experiments, Bowman reached the MYP stage close to the AP stage in only two instances, indicating that the MYP stage can be different from the previously proposed AP stage (Appleyard *et al.*, 1982). In fact, we demonstrate that morphological changes of the inflorescence meristem (IM) during spikelet initiation and growth are highly linked to reaching the MYP stage and the cessation of spikelet initiation. This method for identifying the MYP stage was termed ‘spikelet stop’ (SS) as it axiomatically defines ‘spikelets at the end of spikelet initiation’. Furthermore, by applying the SS method for the identification of the MYP stage and the MYP, we tracked the developmental progression following the Waddington scale (Waddington *et al.*, 1983) and the number of spikelets on the main culm spike in a panel of two- (17) and six-rowed (10) genotypes grown in the greenhouse and the field. Our findings from this panel clarify unambiguously that the MYP stage can occur at various developmental stages and that it depends on genotypes and growth conditions. We also observed that the timing/duration and the stage reaching MYP might determine the GN of the main culm spike, at least in two-rowed types. Finally, this study provides an appropriate methodology for the identification of the MYP stage in barley spikes.

## Materials and methods

### Plant materials and growth conditions

We conducted one greenhouse and one field experiment using a panel of 27 barley accessions chosen from a worldwide collection (Alqudah *et al.*, 2014). The field study was conducted at the Leibniz Institute of Plant Genetics and Crop Plant Research, Gatersleben, Germany (51°49′23″N, 11°17′13″E, altitude 112 m) from March to August 2017 and in the greenhouse from July to December 2017. For both the experiments, we followed similar sowing and pre-treatment protocols. Grains were sown on 96-well trays and grown under greenhouse conditions (photoperiod, 16 h/8 h, light/dark; temperature, 20 °C/16 °C, light/dark) for 2 weeks. The plants were then vernalized at 4 °C for 4 weeks and subsequently hardened in the greenhouse for a week. Following acclimatization, plants were directly transplanted in silty loam soil for the field experiment; however, in the greenhouse experiment (photoperiod, 16 h/8 h, light/dark; temperature, 20 °C/16 °C, light/dark), plants were potted in a 9 cm pot (9×9 cm, diameter×height). For the field experiment, we followed a single plot per genotype design, in which each plot had eight rows with a 15 cm distance between the rows. The rows are 0.8 m long, wherein we sowed five plants per row. Details of the selected barley panel and their field growth conditions are given in Supplementary Tables S1 and S2, respectively.

The series of experiments on cultivar Bowman were performed in the greenhouse and climate chamber. The growth conditions and different pot sizes of the experiments are given in Supplementary Table S3. In all the greenhouse and climate chamber experiments, plants were grown in pots that contain two parts of autoclaved compost, two parts of ‘Rotes Substrat’ (Klasmann-Deilmann GmbH, Germany), and one part of sand. We followed the standard practices for irrigation, fertilization, and control of pests and diseases in these experiments.

### Methods of phenotyping the traits

All traits were measured only from the main culm of a barley plant due to its higher phenotypic stability across growth conditions and its major contribution to the final grain yield (Cottrell *et al.*, 1985; Elhani *et al.*, 2007). In all Bowman spikelet-tracking experiments, plants were randomly selected, and spikes were dissected out almost every alternate day. In the spikelet-tracking experiments with the 27 accessions, random plants were dissected every 2–3 d or more, depending on their developmental rate. Dissection of spikes was performed according to the methods described in Kirby and Appleyard (1984). Different spike developmental stages were identified by following the description provided earlier (Waddington *et al.*, 1983; Kirby and Appleyard, 1984). For every stage of a spike, the decimal code suggested by Waddington *et al.* (1983) was allocated following the letter ‘W’ (Waddington). A decimal code was assigned to a spike when the specific stage was found in a minimum of three or a maximum of four consecutive nodes of a spike (Waddington *et al.*, 1983; Kirby and Appleyard, 1984). These nodes are always the most developed nodes of a spike (Kirby and Appleyard, 1984) and may be found close to the spike base, two to three nodes above the spike base, or in the center of a spike.

For the potential spikelet number (PSN) of a spike, differentiated spikelets and undifferentiated spikelet ridges (usually found at the base and tip of a spike) were counted (Appleyard *et al.*, 1982). Generally, barley forms three spikelets at every rachis node (Bonnett, 1935, 1966; Komatsuda *et al.*, 2007; Koppolu *et al.*, 2013), so the numbers of undifferentiated spikelet ridges were multiplied by three. The number of ridges developed on a spike was considered as the spikelet ridge number (SRN), and the final spikelet number (SN) and GN of a spike were enumerated after physiological maturity. Growing degree days (GDDs) or thermal time was calculated from the average maximum and minimum air temperature of a day with the base temperature subtracted (McMaster and Wilhelm, 1997). We considered 0 °C as the base temperature for barley (Gallagher *et al.*, 1976). GDDs and PSN/SRN were taken from three plants, while SN and GN were from six plants.

### Data analysis

The data were analyzed using the Prism software, version 8.4.2 (GraphPad Software, LLC), and outliers were detected by the ‘ROUT’ method (Motulsky and Brown, 2006). Mean value comparison of different traits was made with either multiple Student’s *t*-tests or unpaired Student’s *t*-test (parametric). The false discovery rate approach of the two-stage linear step-up procedure of Benjamini, Kreier, and Yekutieli (Q=5%) (Benjamini *et al.*, 2006) was used to calculate the significance of Student’s *t*-tests. For traits such as the Waddington scale and GDDs at MYP, a two-way ANOVA with Tukey’s multiple comparison test (α=5%) was used to identify the significance of the mean values. For the SRN, a one-way ANOVA with Tukey’s multiple comparison test (α =5%) was used to identify the significance of the mean values. All the replicates of a genotype were analyzed individually and without assuming a consistent standard deviation. We identified the breaking point (MYP stage) by fitting the SRN data with the logistic growth model (Kucharavy and De Guio, 2015). Linear regression was done using the appropriate dependent (*Y* values) and independent (*X* values) traits. The 95% confidence intervals were identified for every linear regression and plotted as confidence bands along with the ‘goodness of fit’ line.

## Results

### The spikelet/floret initiation and growth pattern of barley

**Fig. 1.**
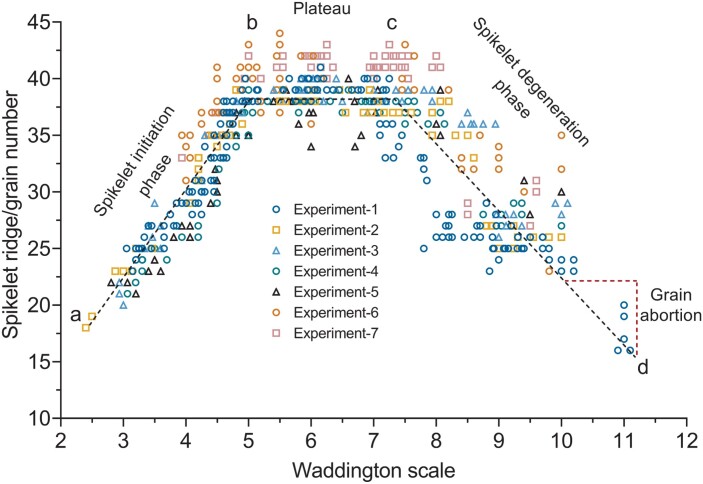
The spikelet/floret initiation and growth pattern of barley. Spikelet initiation and growth pattern of a barley cv. Bowman analyzed in seven different experiments is shown. It shows that the pattern has three phases—the first phase (from point ‘a’ to ‘b’) indicates the increase of spikelet ridge number during the spikelet initiation phase; the second phase (from ‘b’ to ‘c’) is a plateau during which the number of spikelet ridges did not change; and the last phase (from ‘c’ to ‘d’) displays the spikelet degenerative phase and grain abortion phase. The breaking point ‘b’ marks the end of the spikelet initiation phase or the stage of maximum yield potential (MYP). In contrast, point ‘c’ is the initiation of the spikelet abortion phase. Each stage may have data from 1–9 different plants.

We conducted seven experiments (variable pot sizes and growth conditions) using the two-rowed barley cv. Bowman, in which spikelet ridges were counted from the glume primordium stage (W2.5) until pollination (W10.0) (Waddington *et al.*, 1983). Additionally, we counted the final GN (only in experiment 1) of main culm spikes after harvest (considered as W11.0) and plotted the values together with the SRN (Fig. 1). Here, we counted every rachis node with differentiated and undifferentiated spikelet primordia and regarded them as ‘spikelet ridges’. The values of spikelet ridges and grains exhibited a pattern that shows three distinct phases of spikelet initiation and growth. The entire pattern starts from point ‘a’ and ends at point ‘d’ (Fig. 1). In addition to these two stages (start and end), there are two breaking points, ‘b’ and ‘c.’ The phase between moment ‘a’ and ‘b’ shows a steady increase of SRN produced from ~W2.5 to ~W5.0 (Fig. 1) and represents the spikelet/floret initiation phase (Gallagher, 1979; Appleyard *et al.*, 1982). Following the initiation phase, the period between the breaking points ‘b’ and ‘c’ is a plateau (Appleyard *et al.*, 1982), during which the SRN is generally unchanged (Fig. 1). While new spikelet initiation has stopped, spikelets and spikelet ridges formed during the initiation phase continue to grow and differentiate during the plateau phase. The subsequent final phase starts at point ‘c’ and ends at point ‘d’, displaying the gradual decrease of SRN due to spikelet abortion; thus, it is considered the spikelet degeneration phase (Fig. 1). Notably, at the first breaking point (‘b’), a spike bears the maximum SRN that is already equivalent to the MYP, denoting the end of the spikelet initiation phase. Afterwards, the SRN remains similar until the second breaking point (‘c’). At the same time, immediately after this stage (plateau), the spikelet/floret abortion process is initiated, which continues until (or around) the pollination stage (W10.0) (Waddington *et al.*, 1983; Kirby and Appleyard, 1984) (Fig. 1). After pollination, there can still be a reduction in the spikelet/grain number; however, here, it is often due to the deficiency of grain setting that leads to grain abortion (Gallagher, 1979) (Fig. 1). Thus, from our results, it is evident that barley spikelet/floret initiation and growth follow a typical pattern that was reported previously (Gallagher, 1979; Appleyard *et al.*, 1982), and include a spikelet generation phase followed by a period of no further spikelet initiation (plateau), which in turn is followed by the spikelet degeneration phase.

### Spikelet initiation arrest marks the MYP stage, and it can vary depending on growth conditions

**Fig. 2.**
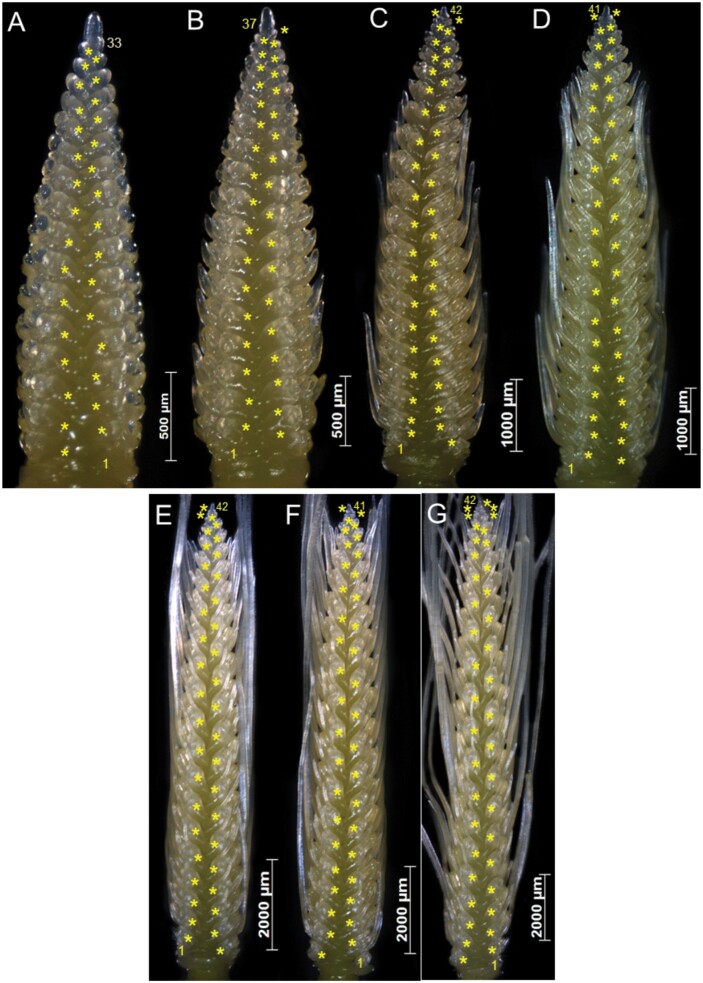
Arrest of spikelet initiation marks the maximum yield potential (MYP) stage in barley spikes. A representative spike of stages from W4.0 to W7.0 and their number of spikelet ridges are shown (A–G) from experiment 7. The number of spikelet ridges steadily increased from W4.0 to W5.0. There were 33 ridges at W4.0 (A) and this increased to 37 in W4.5 or the awn primordium (AP) stage (B). The ridge number increased after the AP stage to 42 in W5.0 (C). After stage W5.0, the number of ridges was not drastically elevated until W7.0 (D–G). These data clearly demonstrate that spikelet initiation stopped at W5.0. From these images, it thus becomes evident that the MYP stage was at W5.0 and not at W4.5 (AP). W, Waddington scale.

**Fig. 3.**
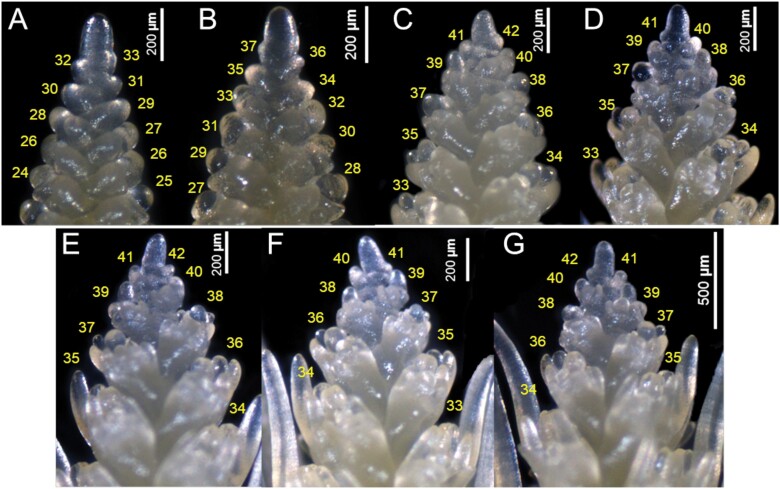
Arrest of spikelet initiation marks the maximum yield potential (MYP) stage in barley spikes. This figure displays the apices of the representative spikes of stages W4.0 to W7.0, shown in Fig. 2. The spike apex or the inflorescence meristem (IM) of W4.0 is shown in (A). It is smooth and has a rounded tip. The IM of W4.5 appeared similar to that of W4.0 (B), while the IM of W5.0 was deformed, and it lost the rounded tip (C). Interestingly, the number of spikelet ridges proliferated only until W5.0, which coincided with the IM deformation. This suggests that the IM lost its activity before W5.0, resulting in the arrest of spikelet ridges. After W5.0, the IM of consecutive stages (W5.5–W7.0) (D–G) never regained the rounded tip; instead, their structures were further deformed, and they became translucent. Thus, this palette of images indicated that W5.0 is the MYP stage. W, Waddington scale.

**Fig. 4.**
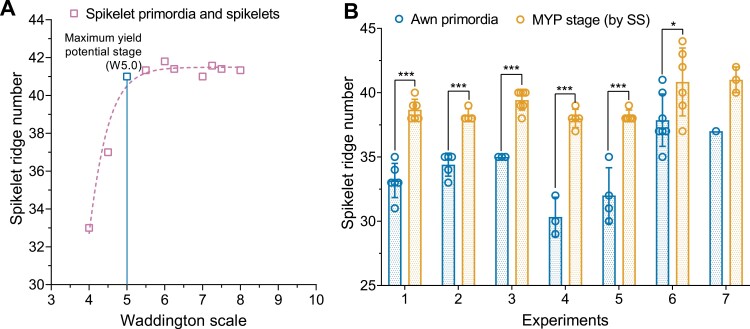
The maximum yield potential (MYP) stage can vary depending on growth conditions. An example of the identification of the MYP stage from experiment 7 is shown in (A). Here, the graph’s breaking point is the MYP stage of the spike. In (B), we show the comparison of spikelet ridge numbers counted at the awn primordium stage (AP, W4.5) and MYP stage (by spikelet stop, SS) from all seven experiments. The graph shows that in almost all the experiments (except experiment 7), the spikelet ridge number counted at the AP stage is significantly lower than at the MYP stage. Due to insufficient replicates of the AP stage in experiment 7, the significance analysis was not performed. Data in (A) are the mean values of spikelet ridge number counted at various developmental stages; with the exception of stages W4.0 and W4.5 (AP) (one replicate), all other stages have 3–7 replicates. The breaking point was identified by fitting the data with the logistic growth model. Data in (B) were analyzed by multiple Student’s *t*-tests with false discovery analysis of Benjamini, Kreier, and Yekutieli with the *Q* value of 5%; with the exception of experiment 7, all other experiments have 3–7 replicates; *P<0.05; ***P<0.001. Replicates are shown as circles and error bars are the SD. W, Waddington scale.

The barley spike belongs to the indeterminate type of inflorescences that develop spikelet primordia in acropetal succession without forming a terminal spikelet (Bonnett, 1935, 1966). In Fig. 2, we display the young Bowman spikes (immature inflorescences) (from experiment 7) of stages W4.0–W7.0, showing the pattern of spikelet ridge development. To this end, we scored the carpel development of the corresponding stages (from W4.5 to W7.0) in Supplementary Fig. S1. In W4.0, the total number of spikelet ridges was 33 (Fig. 2A), which was increased to 37 at W4.5 (Fig. 2B), and then escalated to 42 at W5.0 (Fig. 2C). Notably, after W5.0, the SRN was not drastically changed during at least four more consecutive stages, namely until W7.0 (Fig. 2D–G). Here, the culmination of SRN at W5.0 indicated the arrest of spikelet initiation as well as the loss of IM activity. The gradual loss of the meristematic potential of the IM was evident from the images of spike apices from stage W4.0 to W7.0 (Fig. 3). At W4.0, the IM had a rounded tip (Fig. 3A) with a slightly reduced size at W4.5 (Fig. 3B). Intriguingly, the IM lost its rounded tip at ~W5.0 and modified it to an oblique shape (Fig. 3C). Subsequently, the IM never regained its smooth shape after W4.5 (Fig. 3B); instead, it became tapered in the following stages (Fig. 3D–G). The structural alteration of the IM (Fig. 3C) and the cessation of spikelet ridge formation (Fig. 2C) at ~W5.0 indicated that spikelet initiation was arrested at this stage, and thus stage W5.0 specified the MYP stage of cv. Bowman. Because the MYP stages indicate the stage at which spikelet initiation stops, we termed the method for identifying the MYP stage by definition ‘spikelet stop’ (SS). We also verified the MYP stage of experiment 7 by fitting the data with a logistic growth model (Kucharavy and De Guio, 2015) and spotted the first breaking point of the spikelet initiation curve at W5.0 (Fig. 4A). Additionally, we compared the SRN between the AP stage and MYP stage (identified by the SS method) from all our Bowman experiments. We found an elevation of SRN by 3–8 ridges (at the defined MYP stage across experiments), again indicating that the MYP stage can be different from the AP stage (Fig. 4B). Only in experiment 1 was the MYP stage (~W4.9) close to the AP stage (W4.5) (Supplementary Fig. S2A); however, in this experiment, the SRN is also significantly higher than at the AP stage (Fig. 4B). Thus, our spikelet-tracking studies using the cultivar Bowman unambiguously showed that arrest of spikelet initiation marks the MYP stage, which could be different from the AP stage (W4.5), and that the MYP stage varies depending on growth conditions (Supplementary Fig. S2B).

### The MYP stage is influenced by genotypic and environmental variation

**Fig. 5.**
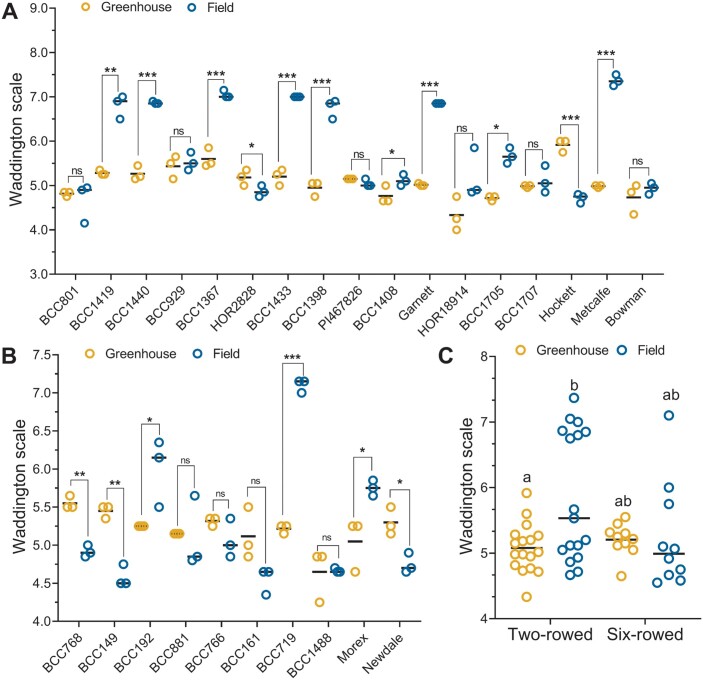
Maximum yield potential (MYP) stage is influenced by genotypic and environmental variation. The MYP stage of 17 two-rowed (A) and 10 six-rowed (B) barley genotypes grown in the greenhouse and field plotted according to the Waddington scale. A comparison of the MYP stage mean value of the two- and the six-rowed panel is shown in (C). In the greenhouse, 16 out of the 17 two-rowed and all the six-rowed types reached the MYP stage between W4.5 and W5.5. However, in the field, eight genotypes from the two-rowed and three from the six-rowed genotypes reached the MYP stage relatively late between W6.0 to W8.0. Interestingly, one of the two-rowed genotypes, HOR2828, and three six-rowed genotypes reached their MYP stage earlier in the field. Each genotype was represented by three different plants in both the greenhouse and field experiments. W, Waddington scale. Data in (A) and (B) were analyzed by multiple Student’s *t*-tests with false discovery analysis of Benjamini, Kreier, and Yekutieli with the *Q* value of 5%; *P<0.05; **P<0.01; ***P<0.001; ns, non-significant. Data in (C) were analyzed by a two-way ANOVA with Tukey’s multiple comparison test (α =5%). Different letters denote the statistical difference of adjusted *P*<0.05.

**Fig. 6.**
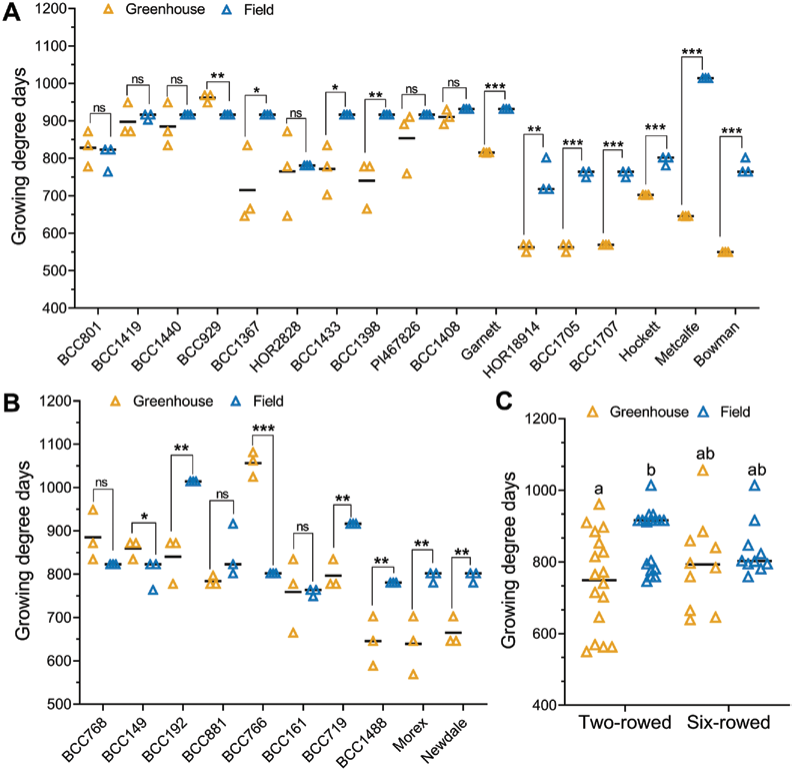
Time to reach the maximum yield potential (MYP) stage is influenced by genotypic and environmental variation. We show the variation of growing degree days (GDDs) to get to the MYP stage of 17 two-rowed (A) and 10 six-rowed (B) barley genotypes grown in the greenhouse and field. A mean value comparison of GDDs to get to the MYP stage of both the panels is shown in (C). In the greenhouse, both the panels attained the MYP stage between 550 and 1000 GDDs, while in the field this was from 750 to 1050 GDDs. Ten two-rowed and five six-rowed genotypes took more GDDs to reach the MYP stage in the field than in the greenhouse. A two-rowed genotype BCC929 (A) and a six-rowed genotype BCC149 (B) made it to the MYP stage earlier in the field than in the greenhouse. Each genotype was represented by three different plants in both the greenhouse and field experiments. Data in (A) and (B) were analyzed by multiple Student’s *t*-tests with false discovery analysis of Benjamini, Kreier, and Yekutieli with the *Q* value of 5%; *P<0.05; **P<0.01; ***P<0.001; ns, non-significant. Data in (C) were analyzed by a two-way ANOVA with Tukey’s multiple comparison test (α =5%). Different letters denote the statistical difference of adjusted *P*<0.05.

Based on the results from the Bowman spikelet-tracking experiments, we applied the SS method for the MYP stage identification in two experiments performed in the greenhouse and field with a panel (27 accessions) of two- and six-rowed genotypes (Supplementary Table S1). In the greenhouse, both row types had a narrow window for attaining the MYP stage, namely as early as ~W4.3 to later stages of ~W6.0 (Fig. 5). In the field, the early MYP stage (~4.5) was not fundamentally different from that in the greenhouse; however, MYP stages were delayed to ~W7.3 in some accessions (Fig. 5), indicating that soil, light, and temperature affected their development. Six two-rowed (BCC801, BCC929, PI467826, HOR18914, BCC1707, and Bowman) and four six-rowed (BCC881, BCC766, BCC161, and BCC1488) genotypes reached the MYP stage at similar stages (i.e. non-significant difference) in both the growth conditions (Fig. 5A, B). In the field, nine two-rowed (BCC1419, BCC1440, BCC1367, BCC1433, BCC1398, BCC1408, Garnett, BCC1705, and Metcalfe) and three six-rowed (BCC192, BCC719, and Morex) genotypes attained the MYP stage significantly later than in the greenhouse (Fig. 5A, B). Interestingly, two two-rowed (HOR2828 and Hockett) and three six-rowed (BCC768, BCC149, and Newdale) genotypes reached the MYP stage significantly earlier in the field than in the greenhouse (Fig. 5A, B). A mean value comparison of both row types for MYP stages from the two growth conditions revealed that the two-rowed types reached the MYP stage significantly later in the field than in the greenhouse due to the behavior of seven (BCC1419, BCC1440, BCC1367, BCC1433, BCC1398, Garnett, and Metcalfe) genotypes. They appeared to be subpopulation within the two-rowed panel (Fig. 5C). Interestingly, among the 27 genotypes grown in two environments, only three six-rowed genotypes (BCC149 and BCC161 in the field; BCC1488 in the greenhouse) attained the MYP stage at or around the AP stage (~W4.5) (Fig. 5B). We also measured the timing to reach the MYP stage in both the experiments by calculating the GDDs. From the analysis of GDDs, it was found that six (BCC1367, BCC1433, BCC1398, Garnett, BCC1705, and Metcalfe) of the nine two-rowed genotypes, which had a higher MYP in the field, also took significantly longer GDDs to reach the MYP stage in the field (Fig. 6A). In six-rowed genotypes, all three (BCC192, BCC719, and Morex) with a higher MYP in the field needed more GDDs to get to the MYP stage (Fig. 6B). The mean value comparison of GDDs in the two growth conditions showed that only in the field was the two-rowed panel separated into two subpanels (Fig. 6C) as with the MYP stage (Fig. 5C). From these results, it becomes clear that the MYP stage can be influenced by genotype and growth conditions.

### Timing and stage of the MYP may determine the grain number in two-rowed barley

**Fig. 7.**
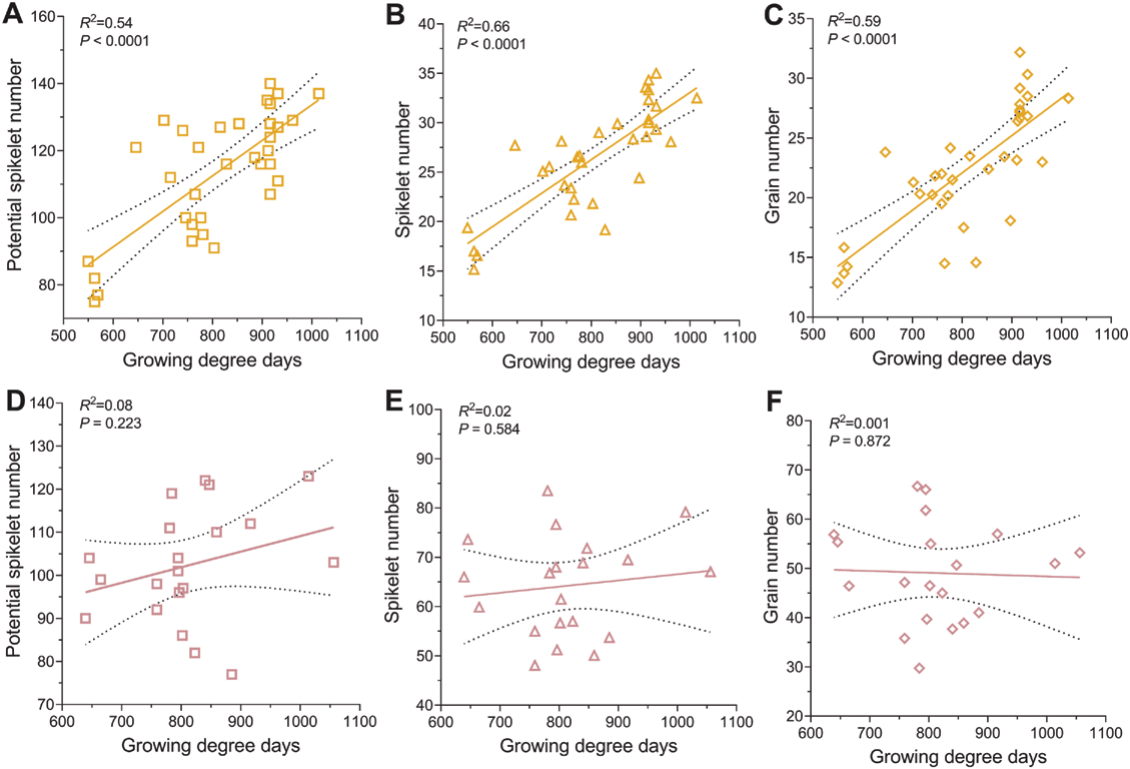
Interactions of timing to the maximum yield potential (MYP) stage and yield components. The growing degree days (GDDs) taken to reach the MYP stage and its interaction with potential spikelet number (PSN), spikelet number (SN), and grain number (GN) of two- (A–C) and six-rowed genotypes (D–F) grown in the greenhouse and field are shown. Two-rowed genotypes that spent more GDDs to get to the MYP stage developed more PSN (A), SN (B), and GN (C), while six-rowed genotypes did not depend on the GDDs to MYP stage for the production of PSN (D), SN (E), and GN (F). GDDs and PSN were taken from three different plants, while SN and GN were from six other plants. The 95% confidence intervals were identified for every linear regression and plotted as confidence bands (black dotted line) along with the ‘goodness of fit’ line.

**Fig. 8.**
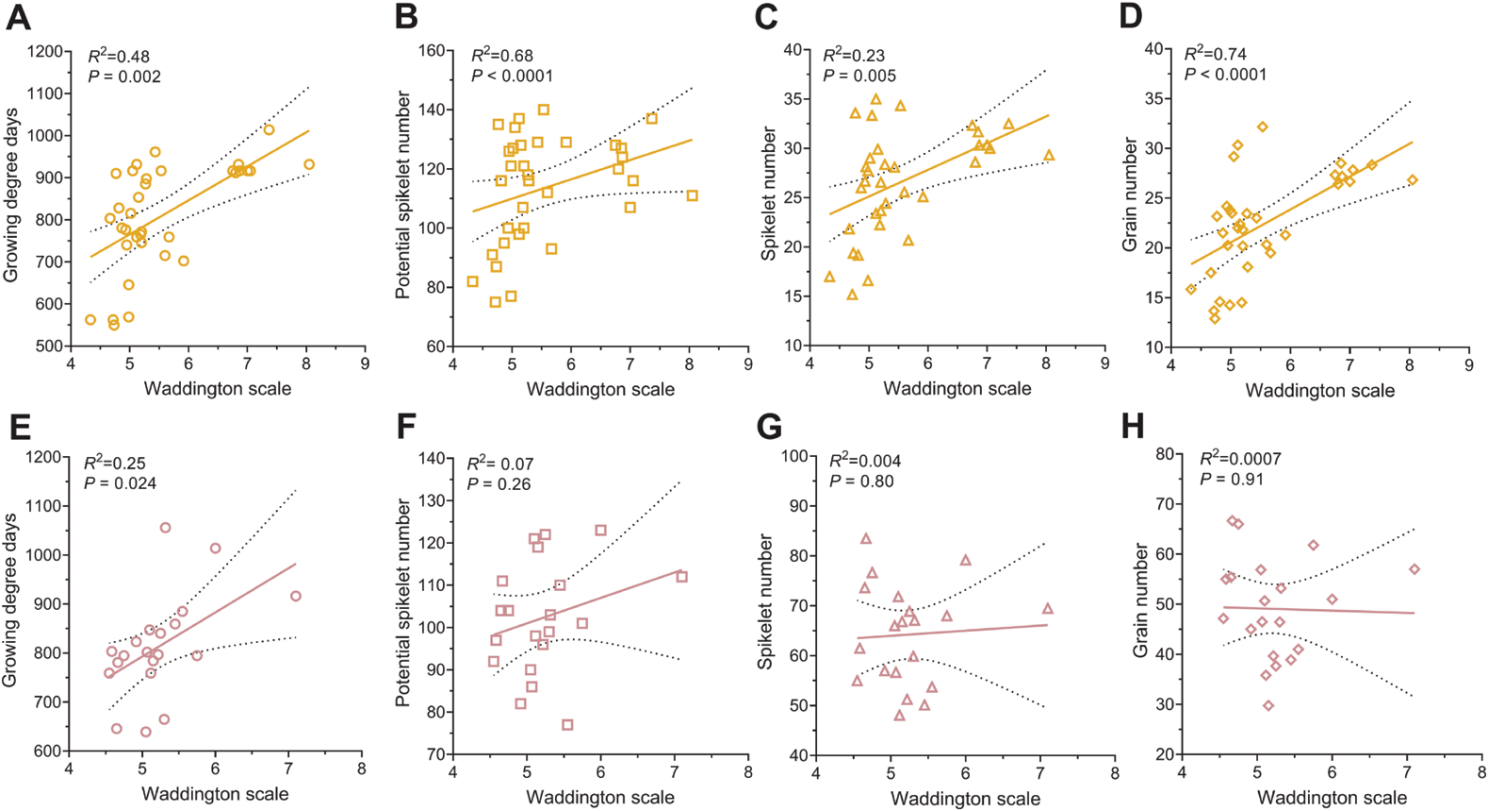
Interactions of the maximum yield potential (MYP) stage and yield components. The MYP stage (as Waddington scale) and its interaction with growing degree days (GDDs) required to reach the MYP stage, potential spikelet number (PSN), spikelet number (SN), and grain number (GN) of two- (A–D) and six-rowed types (E–H) grown in the greenhouse and field are shown. In both row-type panels (greenhouse and field), genotypes reached the MYP stage at a later stage of the Waddington scale, taking more GDDs than earlier genotypes (A and E). Two-rowed genotypes which get to MYP at a later stage of the Waddington scale produced more PSN (B), SN (C), and GN (D), while PSN (F), SN (G), and GN (H) of six-rowed types are independent of the MYP stage. GDDs and PSN were taken from three different plants, while SN and GN were from six other plants. The 95% confidence intervals were identified for every linear regression and plotted as confidence bands (black dotted line) along with the ‘goodness of fit’ line.

To better understand the influence of the timing and stage of the MYP in relation to the final main culm GN, we evaluated the interaction of GDDs to reach the MYP stage and the developmental stage at which the MYP is reached with PSN, SN, and GN of the main culm (Figs 7, 8). For both row types, the SRN at the MYP stage was multiplied by three to obtain the PSN because barley forms three spikelets at every rachis node (Bonnett, 1935; Komatsuda *et al.*, 2007). By combining the results of both growth conditions, it was clear that the two-rowed PSN, SN, and GN are strongly determined by the time taken to get to the MYP stage (Fig. 7A–C). However, the same traits analyzed for six-rowed types appeared independent of GDDs (Fig. 7D–F). Also, the MYP stage (as the Waddington scale) of the two-rowed types significantly influenced the GDDs, PSN, SN, and GN (Fig. 8A–D), while in the six-rowed types, only the GDDs were moderately influenced by the MYP stage (Fig. 8E–H). Thus, our interaction study suggests that the timing and developmental stage at which two-rowed barleys reach their MYP stage may determine their main culm yield potential, whereas this is less valid for six-rowed barleys.

## Discussion

Improvement of spikelet/floret fertility can be a promising avenue to enhance the grain yield of small grain cereals. In general, the spikelet/floret fertility or efficiency of spikelet/floret survival is measured by comparing the MYP with the final spikelet/floret number. Developing a method to identify the barley MYP would pave the way to understanding the genetics and mechanism of spikelet survival in barley.

### The arrest of spikelet initiation specifies the MYP stage in barley, and it can be different from the AP stage

The concept behind the identification of the MYP stage in barley is that it helps to assess how efficiently a plant produces grains from its maximum number of spikelets. To this end, it is crucial to recognize the stage at which a spike possesses its maximum number of spikelets. Several reports have evaluated the rate of barley spikelet development and identified the MYP stage by following the formation of spikelets until the cessation of spikelet initiation (Gallagher *et al.*, 1976; Kirby, 1977; Arisnabarreta and Miralles, 2006*a*; Arduini *et al.*, 2010). However, reports aimed at revealing the genetic variation of barley spikelet/floret fertility (Alqudah and Schnurbusch, 2014) or responses to different treatments and growth conditions (Arisnabarreta and Miralles, 2004, 2006*a*, 2010, 2015) assumed the AP stage as the MYP in all the genotypes and different environmental conditions. Our comprehensive study using the two-rowed cv. Bowman revealed different spikelet initiation and growth phases, as previously shown for cereals (Gallagher, 1979; Appleyard *et al.*, 1982). The end of an increase in spikelet number, or the first breaking point of the graph (Fig. 1), denoted the cessation of the spikelet initiation phase at which a spike possesses its maximum number of spikelets, known as the MYP stage. Interestingly, in all our experiments with the cultivar Bowman, the MYP stage of the spike was significantly different from the AP stage (~W4.5) (Supplementary Fig. S2A, B), in contrast to the findings of a previous report (Appleyard *et al.*, 1982). Similarly, other studies reported that the culmination of spikelet initiation occurred concurrently with stages of stamen primordia (W3.5) (Nicholls, 1963, 1974), W6.0 (shortly after the initiation of the pistil) (Waddington *et al.*, 1983), and after AP (Arduini *et al.*, 2010). In wheat, a study conducted with 12 genotypes reported that the MYP stage consistently occurred during the green anther (GA) stage (Kirby and Appleyard, 1984) among the genotypes, but was similar in the tiller removal treatment (Guo and Schnurbusch, 2015). However, a closer look at the data indicated that in three instances of detillered plants, the MYP stage and the number of florets were significantly different from the GA stage (table 3 in Guo and Schnurbusch, 2015), somewhat underestimating the maximum floret number most likely due to a treatment effect (Supplementary Fig. S3). The above studies suggest that, regardless whether in wheat or barley, researchers should be cautious when analyzing the MYP stage in a limited number of genotypes because most probably the difference in the MYP stage is subtle and/or found only in a few genotypes. Our MYP stage comparison study with the panel (27 genotypes) of two- and six-rowed barley types clearly shows that the MYP stage can be attained from ~W4.0 to ~W7.5 (Fig. 5) by different genotypes in variable growth conditions. Furthermore, tracking the spike apex during early spike development revealed that the IM lost its meristematic activity, visualized by its deformed dome at W5.0 (Fig. 3C) as reported previously (Kirby, 1977). All these findings demonstrate that, in barley, spikelet initiation arrest (i.e. SS) marks the MYP stage, which can be different from the AP stage.

### MYP stage is plastic and may influence the GN of two-rowed barleys

Our extensive studies on Bowman spikelet initiation and growth and the comparison of MYP stages on a selected panel exemplified that the MYP stage is influenced by the genetic variation and different growth conditions. Furthermore, these studies also indicated that SRNs were significantly different in the AP and MYP stage (identified by SS) (Fig. 4B; Supplementary Fig. S4). Intriguingly, the mean SRN counted at the MYP stage (identified by SS) of all the seven Bowman experiments showed a narrow range, 38–41; however, a few experiments have similar MYP, and some are significantly different from each other (Supplementary Fig. S2C). Similarly, the MYP comparison study on the selected panel showed that 12 out of 27 genotypes had a similar SRN in both the growth conditions (Supplementary Fig. S5). Interestingly, four of the above 12 genotypes and six more genotypes (10 in total) had a similar spike developmental scale when they were at the MYP stage (Fig. 5A, B). The above results also implied that 15 and 17 (out of 27) genotypes had significantly different SRN and developmental scales, respectively, when they got to the MYP stage (Supplementary Fig. S4; Fig. 5A, B). A few studies using a limited number of barley genotypes (4–10) claimed that the maximum number of spikelet primordia was not affected due to lower nitrogen supply (Arisnabarreta and Miralles, 2004), shading (Arisnabarreta and Miralles, 2008*b*), and different growth seasons (Arisnabarreta and Miralles, 2006*a*). However, there are also studies with a few accessions (3–6) that reported that the MYP varies among different accessions and sowing dates (Arduini *et al.*, 2010; Digel *et al.*, 2015). We also observed that the MYP stages of two- and six-rowed types showed distinct interactions between the growth conditions. Two-rowed genotypes reached their MYP at similar stages in two growth conditions, whereas six-rowed genotypes had different stages (Supplementary Fig. S6). This could be due to the genotypic variation found in our panel or that the spikelet initiation pattern in the main culm of two-rowed genotypes is more stable than in six-rowed types. Notably, we found the MYP of our panel was always detected after the first node in the main culm in both growth conditions (Zadoks *et al.*, 1974). One study also reported that a few barley genotypes reached their MYP around this stage (Arduini *et al.*, 2010). Furthermore, our GDDs analysis of the MYP stage on the selected panel revealed that analogous to the MYP and its developmental stage, there were two groups: one group (nine genotypes) that did not show a significant difference in GDDs between the growth conditions; and the other group (18 genotypes) that had significant differences (Fig. 6). Based on our findings and the above reports, we propose that the MYP stage, maximum SRN, and GDDs to MYP stage are primarily dependent on the genotypic variation and environmental conditions. Our regression analysis of GDDs to MYP and the MYP stage with the yield potential traits on the selected panel suggests that timing to reach the stage of MYP in two-rowed genotypes may influence the GN of the main culm (Figs 7, 8). A similar finding was reported for two-rowed genotypes that had different alleles of *Ppd-H1*, where, under long-day conditions, introgression lines with a mutant *ppd-H1* took a longer time to reach W3.5 and produced more grains per spike compared with the barley lines with the wild-type allele (Digel *et al.*, 2015). In contrast, in six-rowed types, except for a weak interaction between GDDs and MYP stage (Fig. 8E), there was no association of either the MYP stage or GDDs with yield potential traits (Figs 7D–F, 8F–H). For six-rowed types, it could be interpreted as if yield potential traits are independent of the MYP stage and GDDs, or that there was less variation in our selected panel of six-rowed genotypes for the MYP stage and GDDs. Therefore, we propose that the associations of the MYP stage and GDDs with GN must be verified with a larger panel of accessions representing the available variations in two- and six-rowed barley. Thus, our study’s findings suggest that the MYP stage and its associated traits are plastic, and it may influence the yield potential in barley.

### Conclusion

Identifying the MYP stage and finding the maximum number of spikelets of a barley spike are two different tasks. Determining the MYP stage requires tracking the spikelet initiation from early development; therefore, it is almost impossible to do that for a larger panel (several hundred) of barley accessions. From the knowledge gained from this study, as well as from previous publications, we therefore recommend a few key steps to identify the MYP of a barley spike easily. An initial spike dissection (shown in Kirby *et al.*, 1984) can be performed when the first node (Zadoks *et al.*, 1974) is detected on the main culm to verify whether the spike has reached its MYP. However, for a larger panel of accessions, one would not get the MYP for all genotypes at the first node stage. Thus, it may be necessary to go for another one or two dissections. We suggest that the second dissection could be after a week or less, depending on the rate of development. If, at the second dissection, the spike has already entered the spikelet degeneration (abortion) phase, it would still be possible to count the spikelet ridges, because during early stages of abortion the spike apex does not disintegrate (Supplementary Fig. S7). To verify whether the spike has already reached the MYP, the change of the IM shape (tapered IM) (Fig. 3) can be used as a visible marker. If the spike at the second dissection is not in the MYP stage, a third attempt similar to the second dissection could be tried. Based on our experience from this study, we believe that an early first dissection (around the first node detection) followed by one or two periodic dissections might help to determine the MYP of most of the genotypes. Notably, the plateau stage of spike development may provide an excellent opportunity to assess the MYP of variable genotypes at varying stages.

## Supplementary data

The following supplementary data are available at *JXB* online.

Fig. S1. Carpel development from W4.5 to W7.0.

Fig. S2. Maximum yield potential (MYP) stage can be different from the awn primordium stage.

Fig. S3. Reanalysis of the de-tillered data from Guo and Schnurbusch (2015).

Fig. S4. Maximum yield potential (MYP) stage can be different from the awn primordium stage.

Fig. S5. Maximum yield potential comparison of 27 barley accessions.

Fig. S6. Interaction of maximum yield potential (MYP) stage with growth conditions.

Fig. S7. Spikelet ridges on aborted spike apices.

Table S1. Details of the 27 accessions used in this study.

Table S2. Growth conditions of the field and greenhouse experiments.

Table S3. Growth conditions of the Bowman experiments.

erab342_suppl_Supplementary_Figures

erab342_suppl_Supplementary_Tables

## Data Availability

The data that support the findings of this study are openly available in Dryad Digital Repository at https://doi.org/10.5061/dryad.ffbg79cth; Thirulogachandar and Schnurbusch, (2021).
