# Supplementary material for: ‘Spikelet stop’ determines the maximum yield potential stage in barley
Source: J Exp Bot. 2021 Jul 22;72(22):7743–53. doi: 10.1093/jxb/erab342 (PMC8643653; doi:10.1093/jxb/erab342)
Supplement: erab342_suppl_Supplementary_Figures [file erab342_suppl_supplementary_figures.pdf]

Supplementary figures

‘Spikelet stop’ determines the maximum yield potential stage in barley

Venkatasubbu Thirulogachandar[1*], and Thorsten Schnurbusch[1, 2*]

[1] Independent HEISENBERG Research Group Plant Architecture, Leibniz Institute of Plant Genetics and Crop Plant Research (IPK), Corrensstr. 3 OT Gatersleben, 06466 Seeland Germany.

[2] Institute of Agricultural and Nutritional Sciences, Faculty of Natural Sciences III, Martin Luther University Halle-Wittenberg, 06120 Halle, Germany

*Correspondence:
Thirulogachandar, V. (venkatasubbu@ipk-gatersleben.de)
Schnurbusch, T. (schnurbusch@ipk-gatersleben.de)

ORCIDS
V.T. 0000-0002-7814-5475
T.S. 0000-0002-5267-0677

Figure S1

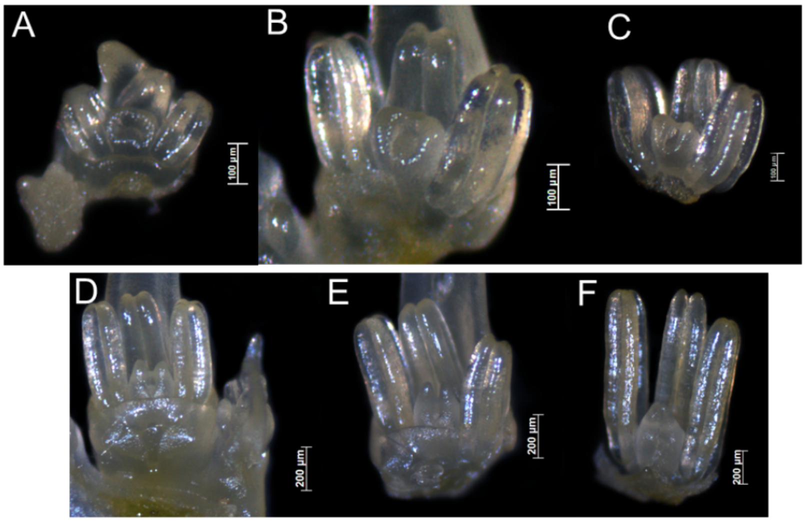

Figure S1: **Carpel development from W4.5 to W7.0.** Development of carpels according to the scale proposed by Waddington et al., 1983 is given in A-F. Figure A indicates the Waddington (W) stage 4.5, B shows W5.0, C displays W5.5, D specifies W6.0, E denotes W6.25, and F discloses W7.0. W-Waddington scale.

Figure S2

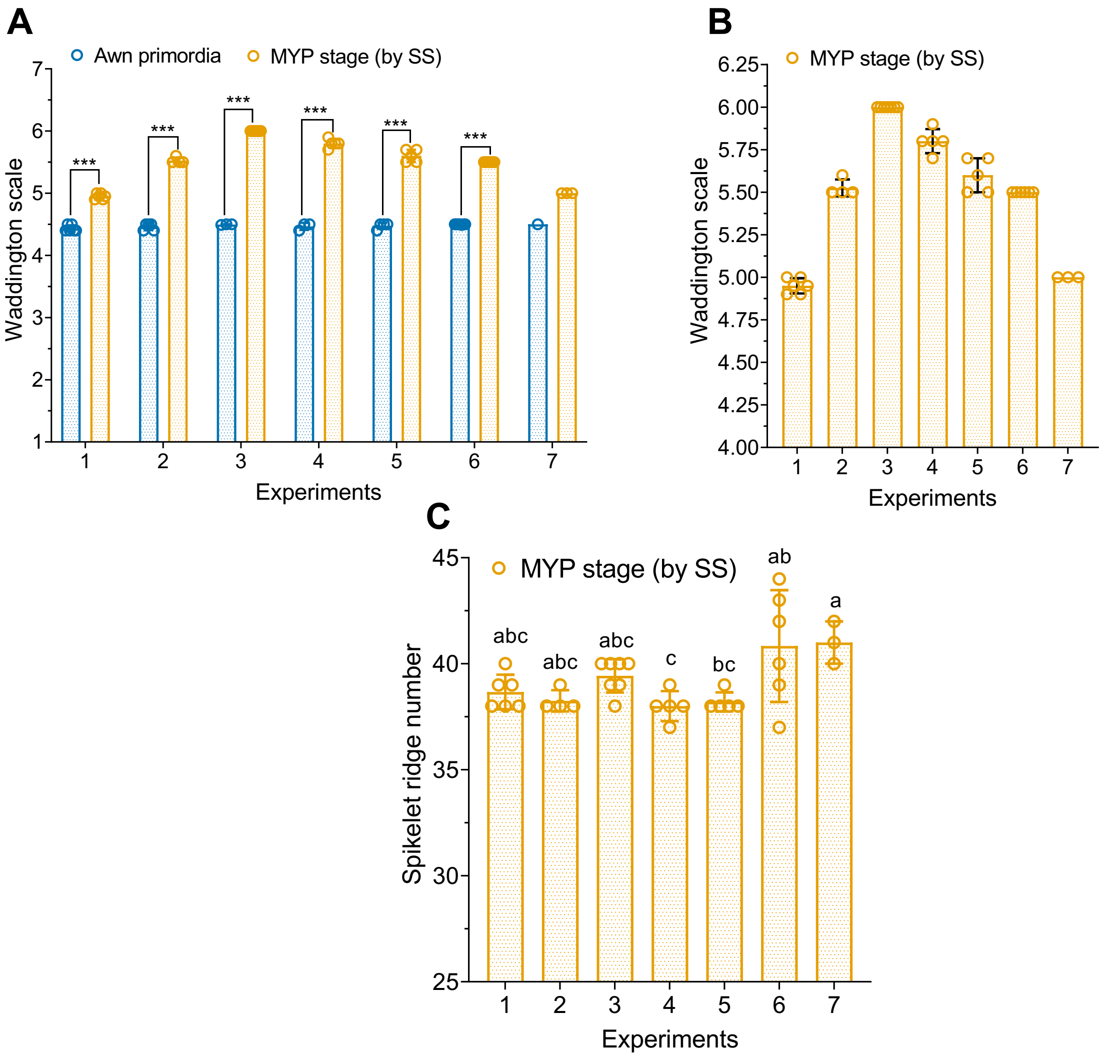

Figure S2: **Maximum yield potential (MYP) stage can be different from the awn primordium stage.** The Waddington scales of the maximum yield potential (MYP) stage (by spikelet stop, SS) and the awn primordium stage (AP) identified in seven experiments are shown in (A), and the Waddington scales of the MYP stage (by SS) found in the seven experiments are shown in (B). The variation of spikelet ridge number at the MYP stage (by SS) in all the seven experiments is shown in (C). Except for experiment 7 that had only one replication for the AP stage, all other experiments had three to seven replications. Data in A were analyzed by multiple Student's *t*-tests with false discovery analysis of Benjamini, Kreier, and Yekutieli with the *Q* value of 5%; ***, $P<0.001$. Data in C were analyzed by a one-way ANOVA with Tukey's multiple comparison test (alpha=5%). Different letters denote the statistical difference of adjusted $P<0.05$. Replicates are shown as circles and error bars are SD.

Figure S3

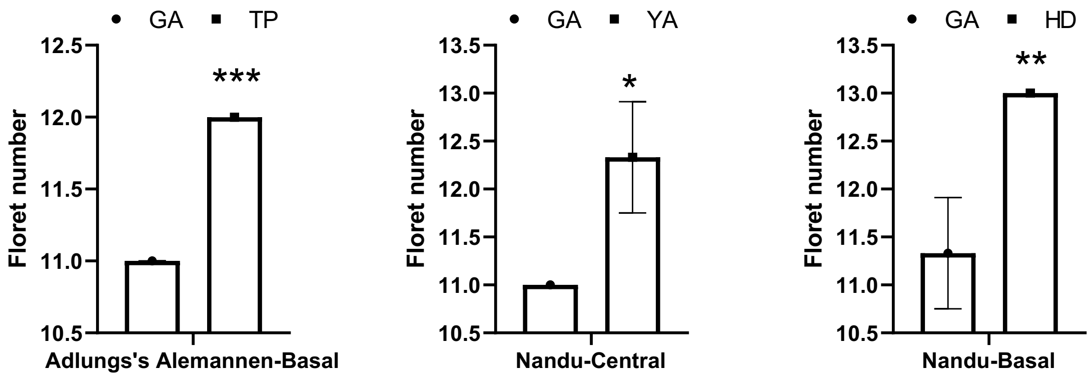

Figure S3: **Reanalysis of the de-tillered data from Guo & Schnurbusch, 2015.** By reanalyzing the table 3 data (de-tillering experiment), we found that in three instances, the maximum yield potential (MYP) stage is significantly different from the GA (green anther) stage. Importantly, this data represents a single spikelet located in various positions (central, basal, or apical). If we extrapolate this discrepancy to the whole spike, it indicates that one might underestimate the MYP of certain genotypes. Data are shown as mean±SD; n=3; Data was analyzed by the Student's two-tailed *t*-test; ***, $P<0.001$; **, $P<0.01$; *, $P<0.05$.

Figure S4

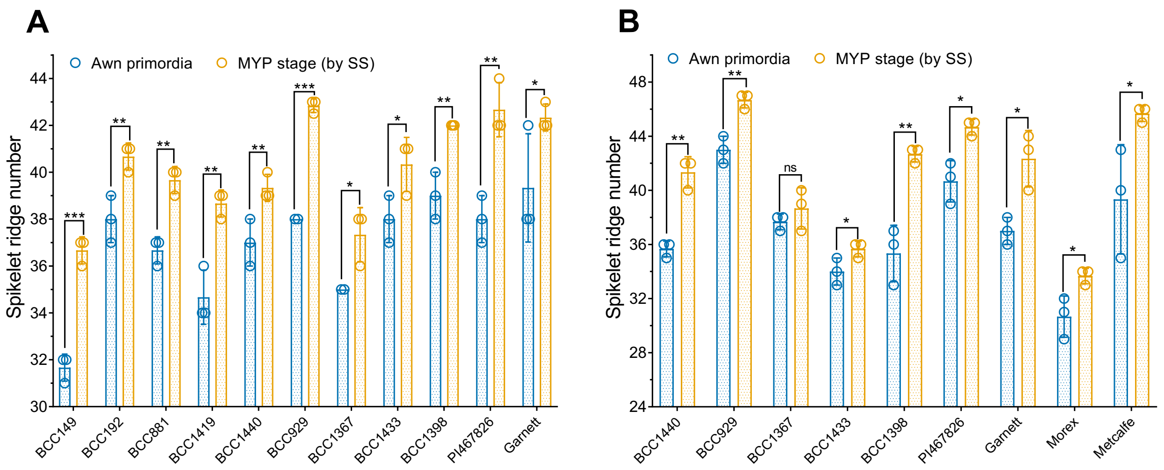

Figure S4: **Maximum yield potential (MYP) stage can be different from the awn primordium stage.** We show the number of spikelet ridges counted for various genotypes at the awn primordium (AP) stage and maximum yield potential (MYP) stage (by spikelet stop, SS) in the greenhouse (A) and field (B). From the 13 genotypes' available data, only one two-rowed type BCC1367 had a similar number of spikelet ridges both in the AP and MYP stage (by SS). Each genotype was represented by three different plants in both the greenhouse and field experiments. Data in A & B were analyzed by multiple Student's *t*-tests with false discovery analysis of Benjamini, Kreier, and Yekutieli with the *Q* value of 5%; *, P<0.05; **, P<0.01; ***, P<0.001. Replicates are shown as circles and error bars are SD.

Figure S5

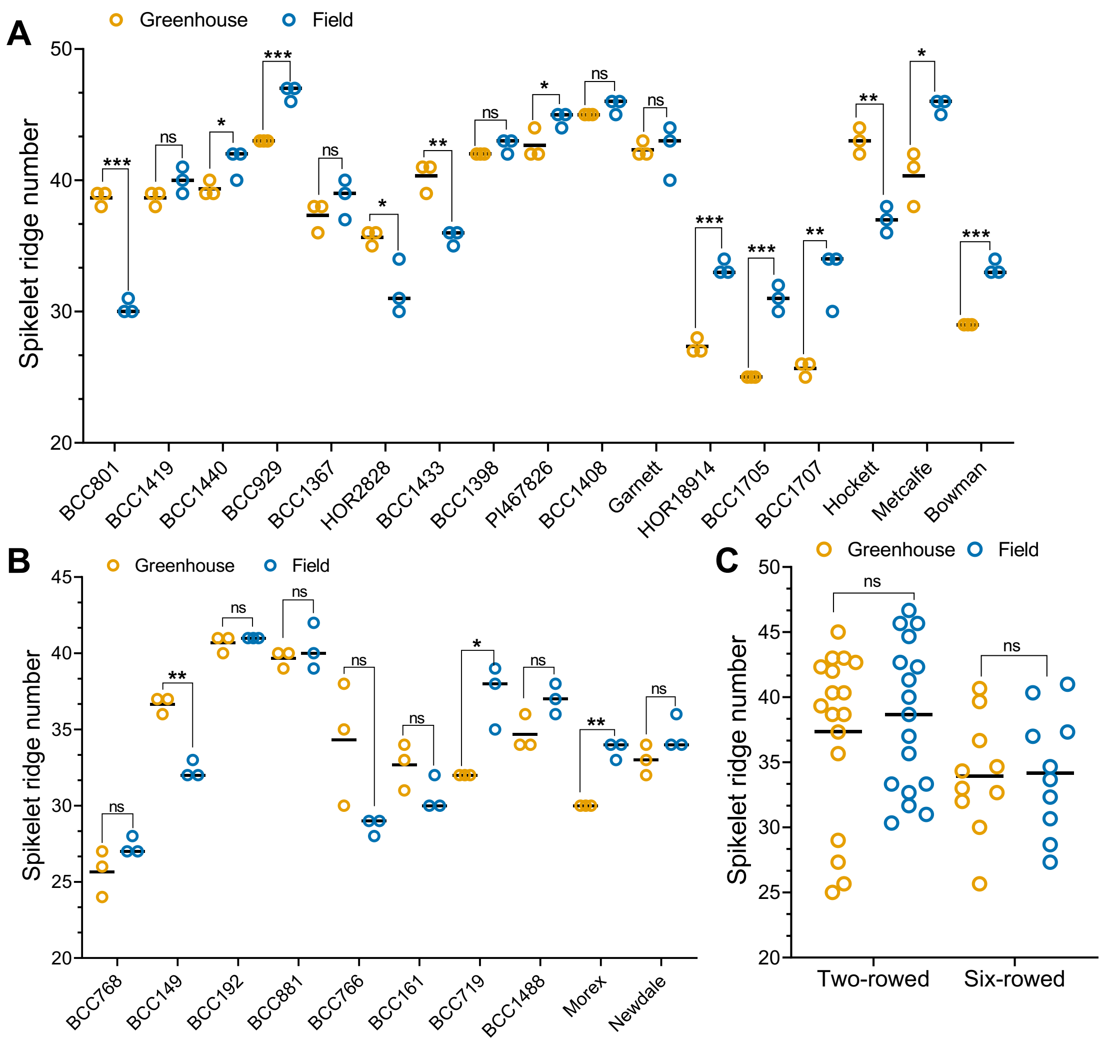

Figure S5: **Maximum yield potential comparison of 27 barley accessions.** We show the variation of maximum yield potential (MYP) as spikelet ridge number (SRN) in 17 two-rowed (A) and ten six-rowed (B) barleys grown in the greenhouse and field. A mean value comparison of the MYP of both the panel is shown in C. Each genotype was represented by three different plants in both the greenhouse and field experiments. Data in A & B were analyzed by multiple Student's *t*-tests with false discovery analysis of Benjamini, Kreier, and Yekutieli with the *Q* value of 5%; *, *P*<0.05; **, *P*<0.01; ***, *P*<0.001; ns, non-significant. Data in C were analyzed by a two-way ANOVA with Tukey's multiple comparison test (alpha=5%); ns, non-significant.

Figure S6

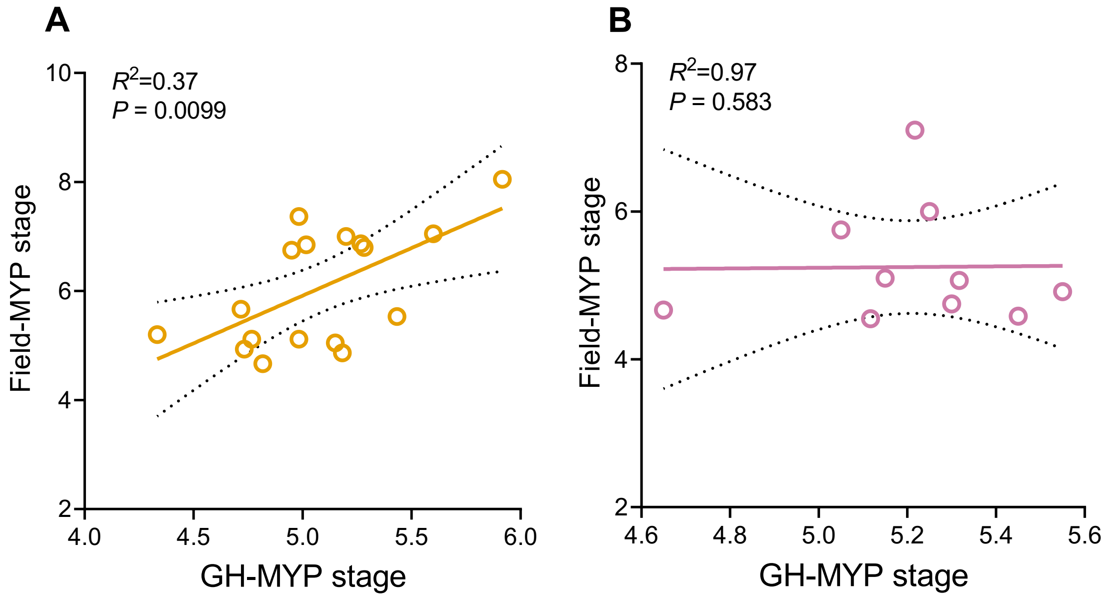

Figure S6: **Interaction of Maximum yield potential (MYP) stage with growth conditions.** The interaction of the greenhouse and field MYP stages of two-rowed (A) and six-rowed (B) are shown. Two-rowed genotypes reached their MYP at similar stages in the greenhouse and field (A), while, six-rowed genotypes' MYP stages are different between the growth conditions.

Figure S7

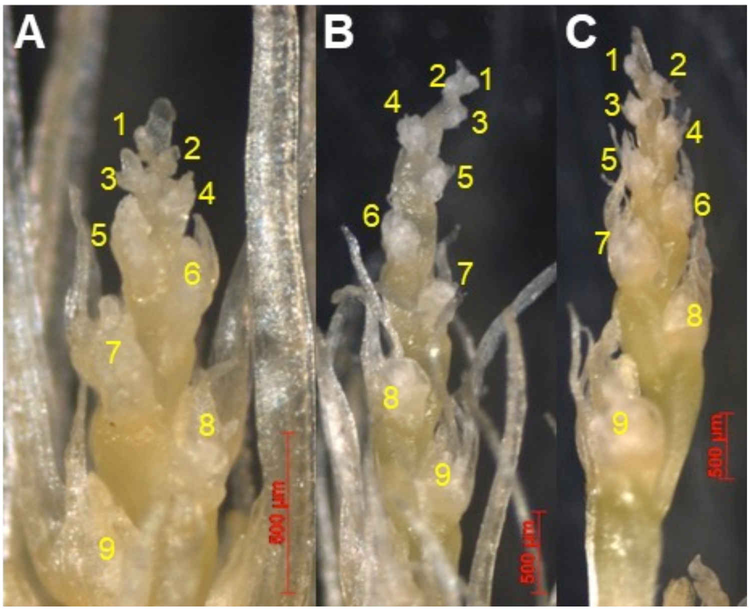

Figure S7: **Spikelet ridges on aborted spike apices.** We displayed examples of aborted spike apices and their counted spikelet ridges in A, B, & C.
